# Cases Illustrative of the Effects of an Impaired Condition of the Blood upon the General System

**Published:** 1852-07

**Authors:** U. P. Golliday

**Affiliations:** Lacon Ill.


ART. II.—*Cases Illustrative of the Effects of an Impaired Condition of the Blood upon the general System.* By U. P. GOLLIDAY, M.D., of Lacon Ill.

IN the May No. of the *Journal* an abstract of the case of Captain Cline was published, which, as I then remarked, was peculiarly interesting from its obscurity, and the very unusual malformation revealed by the autopsy.

I now refer to it again, as illustrative of the effects upon the general system, of an impaired or impoverished condition of the blood.

It will be remembered there was but one kidney, and it was enormously enlarged. This enlargement was not natural, but was a hypertrophied condition of the organ, caused by the absence of the other, it having to perform the functions of both. In time, from being overtasked, it would be incapable of properly eliminating effete matter from the blood, and urea and other excretable matters would remain, and become sources of irritation, more or less impairing the functions of other organs. In this way the liver would become involved, and the mischief would go on with an increasing development, causing local inflammations, resulting in loss of life. His case is additional evidence of the enduring capabilities of nature, for the wonder is, that the integrity of the organism could have been maintained so long a time (39 years,) without any great disturbance of the system; but when it began to take place, the powers of life were directly overwhelmed.

I now proceed to report another fatal case, arising from an almost opposite cause. In Captain C.'s case the excreting functions were defective, and the blood was impaired from the presence of effete matters which should have been thrown off. In the case under consideration, the assimilative organs were probably first in fault, and the blood was impaired from want of those healthy constituents necessary to give it a life-sustaining force. In both cases the results were local inflammations under which life gave way. In the first, unsuspected as it regards the particular organ implicated; in the other, known and understood; in both, obscure as to the extent of the mischief.

Saturday, 10 P. M., March 20, 1852. Called to Mr. John G——, æt. about 55, dark hair and eyes; temperament mixed, bilious predominant. Of a robust, athletic frame, not inclined to corpulency. Occupation, farmer.

He had no pain, seemed easy, and at that time without fever; pulse low and feeble, at times variable, at about 100 to 110. Respiration rather hurried and laborious; a slight cough, so seldom in fact as scarcely to be noticed, and without expectoration. Percussion showed some dullness in the infra-mammary and infra-axillary regions of right side. Respiratory murmur natural, and distinctly audible except in regions above noted.

Whilst raising a barn on the 16th (Tuesday preceding,) a small beam had fallen, striking him on the right postero-lateral surface of the chest, when in a stooping position, not very severe, but enough to throw him to the ground. On the next day (Wed. 17,) he had a chill followed by considerable fever, and had been more or less feverish every day until I was called. He had no pain except under firm pressure, or when taking a very full breath, and then only in the injured side. Had no appetite, and when urged by the family to take food, usually declined, saying he would get up directly and then would; yet he kept his bed from the time of the chill until Saturday evening, when he arose, but soon fainted.

I may here remark, that for some one or two years preceding, he had not had good health; frequent loss of appetite, with various dyspeptic symptoms; was easily fatigued, and unable to endure toil and labor as he had been accustomed to do. Sometimes he complained of a dizziness of the head, and ringing in the ears; but during this time did not think it necessary to call a physician, merely using an occasional gentle aperient.

His case presented some obscurity; but from the slight pain and uneasiness when the lungs were inflated—the hacking cough—the dullness, &c., I regarded the case as one of masked or latent pneumonia, and prescribed the following:

℞—Calomel,

Tart. antimony, *aa.* grs. v

Nit. potassa, grs. xxx.

Divided into ten powders, one to be taken in elm water every four hours. Episp. to side.

No blood was taken, as from the previous history of the case—the time elapsed since the present attack—the condition of the pulse, his general appearance, &c., I believed the attack to be of an asthenic character, and that it arose from a vitiated and impaired condition of the circulation.

March 22. General condition much as when left yesterday morning. More decided pneumonic symptoms, as bloody sputa, pain, &c., had shown themselves, but were again subsiding. Blister had drawn well. Antimony had not caused either nausea or catharsis. Medicine continued at longer intervals, with the following alternate:

℞—Quinine grs. x
Carb. ammonia
Camphor *aa* grs. v
Tart. antimony grs. iij.

Divide into ten powders.

The following to be occasionally substituted for the calomel and antimony powders first given:

℞—Tinct. hyosciami f℥iij
Mel. scill. comp. f℥j
Tinct. Sanguinaria f℥j
Tart. antimony gr. j. Take one teaspoonful.

Wed. 24. At my request Dr. Boal visited Mr. G—— with me. His views corresponded with my own

Pulse more variable, at times intermittent; a slight incoherency of thought and language beginning to manifest itself. Still some dullness in right side; left lung clear. Blister again applied.

Sab. 28. Saw Mr. G—— every day since last date. Incoherency had increased to delirium, with subsultus tendinum, picking the bed clothes, grasping at imaginary objects overhead, or about the bed, and when slumbering (he had not slept since Wednesday) a continual muttering. There had been no evidence of pneumonia since the 25th, when the antimony and nit. potass. had been suspended, and morphine or opium, camphor, quinine, wine, &c., &c., had been used, singly or in combination as circumstances had seemed to require.

The case had now assumed a most critical aspect; but on a careful review of it since the attack, his previous condition for the last year, &c., I felt confirmed in the opinion that the pneumonia of the first few days of his illness, and the incoherency and delirium now present, were not caused by a true inflammatory action, but were the local consequences of a vitiated or impoverished condition of the blood, and that the true indications were—not to deplete, but to strengthen by stimulants and tonics.

With this view, I now directed nourishing food, taken at regular intervals,—brandy, tinct. cinchon. comp., quinine, camphor, opium; the last three combined and given with the tincture of bark every hour and a half or two hours; the brandy as circumstances required.

Tues. 30. Delirium has nearly or quite subsided; his pulse is more regular. No indication of thoracic disease except the persistance of dullness of the right side and a slight cough. Complains of soreness of the throat. On examination, the fauces and roof of the mouth were found to be covered with patches of aphthous inflammation, with intervening spaces of healthy looking membrane. After a slight effort at coughing, portions of matter resembling crusts or scabs from ulcerated surfaces, were thrown up. One I observed was half an inch across.

Medicine continued, only directing wine two parts, brandy one part, instead of brandy alone; and an astringent gargle for the mouth.

Under this treatment he seemed to be gradually improving, appetite returning, and hopes of ultimate recovery seeming to brighten.

April 8. Diarrhœa has set in; the discharges soon became bloody, mixed with mucus, resembling those common to dysentery, but without tenesmus, abdominal soreness, or even pain. Astringent tonics, wine, &c., formed basis of treatment.

April 24. Bowel complaint has continued since last date. Aphthous crusts have again shown themselves in the mouth. Ipecac, sulphas zinci, alum, nit. argent., argenti chlorid, kreasote, opium, kino, preparations of iron, &c., had all been unavailingly used. At my request Dr. L. G. Thompson saw him with me to-day, and suggested infus. serpen., a resume of tinct. cinchon. comp., &c., with injections of fresh butter, tinct. opii, etc.

29. Weaker and evidently sinking. Since last date, infus. serpen. had to be abandoned, and pills of quinine, sulph. ferri and opium were used.

Died 30th. Autopsy, Saturday, May 1st, assisted by Dr. Thompson, 26 hours after death.

Body, emaciated. Lungs—left, sound, excepting that in the apex were some portions of condensed structure resembling old cicatrices. Right, adherent throughout superior antero-lateral portions, the apex quite firmly; adhesions appeared to be of old date; posterior and inferior portions free; structure apparently healthy.

Heart, spleen, and kidneys, natural. Liver somewhat enlarged, but no special evidence of disease. Stomach natural.

The principal ravages of disease were found in the colon and rectum, and involving some twelve or fourteen inches of the lower portion of the ileum. Throughout this extensive tract of intestinal tube, the mucous membrane was inflamed and thickened, presenting numerous patches of ulceration; many ulcers having penetrated through the mucous coat, involving the investing tissues in the consecutive inflammation, and giving the whole inner surface a ragged, honeycomb appearance.

